# Metformin effectively treats *Tsc1* deletion-caused kidney pathology by upregulating AMPK phosphorylation

**DOI:** 10.1038/s41420-020-0285-0

**Published:** 2020-06-15

**Authors:** Yili Fang, Fang Li, Chenyang Qi, Xing Mao, Feng Wang, Zhonghua Zhao, Jian-Kang Chen, Zhigang Zhang, Huijuan Wu

**Affiliations:** 1 Department of Pathology, School of Basic Medical Science, Fudan University, Shanghai, 200032 PR China; 2 Department of Nephrology, Shanghai 6th People’s Hospital, Shanghai Jiaotong University School of Medicine, Shanghai, 200032 PR China; 3 Department of Cellular Biology & Anatomy, Medical College of Georgia, Augusta University, Augusta, GA 30912 USA

**Keywords:** Polycystic kidney disease, Experimental models of disease

## Abstract

Tuberous sclerosis complex (TSC) is characterized by hamartomatous lesions in multiple organs, with most patients developing polycystic kidney disease and leading to a decline of renal function. TSC is caused by loss-of-function mutations in either *Tsc1* or *Tsc2* gene, but currently, there is no effective treatment for aberrant kidney growth in TSC patients. By generating a renal proximal tubule-specific *Tsc1* gene-knockout (*Tsc1*^ptKO^) mouse model, we observed that *Tsc1*^ptKO^ mice developed aberrantly enlarged kidneys primarily due to hypertrophy and proliferation of proximal tubule cells, along with some cystogenesis, interstitial inflammation, and fibrosis. Mechanistic studies revealed inhibition of AMP-activated protein kinase (AMPK) phosphorylation at Thr-172 and activation of Akt phosphorylation at Ser-473 and Thr-308. We therefore treated *Tsc1*^ptKO^ mice with the AMPK activator, metformin, by daily intraperitoneal injection. Our results indicated that metformin increased the AMPK phosphorylation, but decreased the Akt phosphorylation. These signaling modulations resulted in inhibition of proliferation and induction of apoptosis in the renal proximal tubule cells of *Tsc1*^ptKO^ mice. Importantly, metformin treatment effectively prevented aberrant kidney enlargement and cyst growth, inhibited inflammatory response, attenuated interstitial fibrosis, and protected renal function. The effects of metformin were further confirmed by in vitro experiments. In conclusion, this study indicates a potential therapeutic effect of metformin on *Tsc1* deletion-induced kidney pathology, although currently metformin is primarily prescribed to treat patients with type 2 diabetes.

## Introduction

Tuberous sclerosis complex (TSC), a genetic disorder affecting multiple organ systems (including the brain, skin, heart, lungs, and kidneys), is caused by loss-of-function mutations in either *TSC1* or *TSC2* gene^1^. In normal human cells, the proteins TSC1 (also known as hamartin) and TSC2 (also called tuberin), which are encoded by *TSC1* and *TSC2* genes, respectively^2,3^, interact with TBC1D7 to form a functional ternary complex in which TSC2 acts as a selective GTPase-activating protein for the small GTPase, RheB (Ras homolog enriched in brain)^4^. When *TSC1* or *TSC2* gene mutations inactivate the ternary TSC complex, Rheb is released to activate the mechanistic target of rapamycin complex 1 (mTORC1), causing dysregulated cell proliferation. In total, 60–80% of TSC patients suffer from renal manifestations of aberrant cell growth and dysregulated cell proliferation, resulting in renal cysts and hamartomatous renal tumors such as angiomyolipomas^1,5,6^. Such renal lesions can cause kidney enlargement and tubulointerstitial fibrosis, leading to impaired renal function and/or angiomyolipoma-associated hemorrhage^7,8^. Although more than half of TSC patients fail to be diagnosed at the early stage of this disease, with innovative detection technologies, more and more TSC patients are diagnosed with kidney pathology^9^.

Currently, mTORC1 is considered a pharmacological target for the treatment of TSC^7,10^. Unfortunately, mTORC1 inhibitors currently used in the clinic seemed to only have a limited cytostatic effect, as the TSC tumors grew again after mTORC1 inhibitors were discontinued, and long-term mTORC1 inhibition could cause detrimental side effects^11–15^. It is noteworthy that deletion of human *TSC2* and *PKD1* genes results in severe infantile polycystic kidney disease (PKD)—a contiguous gene syndrome^12^. Neither sirolimus (rapamycin) nor everolimus (sirolimus analog) is effective in treating PKD, and these drugs have been reported to cause albuminuria (caused by sirolimus) and leukopenia (caused by everolimus)^16–18^. Moreover, our previous study demonstrated that either pharmacologic inhibition of mTORC1 or genetic deletion of rpS6 phosphorylation exacerbated structural and functional damage of the kidneys in *Tsc1* gene-deleted mice^11^, suggesting that alternative strategies are needed for effective treatment of TSC-associated kidney disease.

AMP-activated protein kinase (AMPK) is involved in multiple physiological regulations^16^. AMPK regulates cell proliferation, apoptosis, and inflammation, in addition to regulation of energy metabolism^19–21^. For instance, modulating AMPK has been demonstrated to be an effective maneuver for treating primary breast cancer, ovarian cancer, and inflammatory bowel disease^22–25^. TSC-associated kidney pathology includes tubulointerstitial fibrosis^11^ and decreased apoptosis^26^. Interestingly, pharmacological activation of AMPK has been demonstrated to be an effective strategy for the treatment of mouse *Pkd1* deletion-induced PKD^21^. Metformin is a potent AMPK activator and well-tolerated drug currently used for the treatment of type 2 diabetes. Metformin has been reported to increase AMPK activity in *Pkd1*^−/−^ cells and prevent renal interstitial fibrosis in a mouse model of unilateral ureteral obstruction (UUO)^27,28^, in addition to ameliorating renal injuries in PKD^21^. Enhancing AMPK phosphorylation with metformin can inhibit the growth and survival of cancer cells by inhibiting protein synthesis and angiogenesis^29,30^, and has been reported for the treatment of cancer^31^. Furthermore, metformin suppresses macrophage infiltration^32^ and inhibits expression of the inflammatory cytokine IL-6^33^. Interestingly, metformin also alleviates lung fibrosis^34^.

To date, however, the effect of AMPK modulation on TSC-associated kidney disease remains unknown. In the present study, we established an animal model of TSC by knocking out mouse *Tsc1* gene in the renal proximal tubule (*Tsc1*^ptKO^), and investigated the effect of metformin on renal pathology and functional changes to evaluate the potential of metformin for the treatment of TSC-associate kidney disease. Utilizing this *Tsc1*^ptKO^ mouse model, we also examined the cellular and molecular mechanisms of action of metformin in treating renal pathology and preserving renal function in TSC.

## Materials and methods

### Reagents and antibodies

Antibodies against β-actin (#66009-1-Ig), IL-1β (#16806-1-AP), IL-6 (#66146-1-Ig), and Fibronectin (#15613-1-AP), as well as goat anti-rabbit (#SA00001-1) and goat anti-mouse (#SA00001-15) secondary antibodies were purchased from Proteintech (Rosemont, IL, USA). Antibodies against TSC1 (#6935), p-AMPK Thr-172 (#2535), AMPK (#5832), p-Akt Ser-473 (#4060), p-Akt Thr-308 (#13038), Akt (#2920), PCNA (#2586), Caspase-3 and Cleaved caspase-3 (#9664), BAD (#9239), BAX (#5023), F4/80 (#30325), CD3 (#99940), CD4 (#25229), and α-SMA (#19245) were purchased from Cell Signaling Technology (Danvers, MA, USA). Rabbit anti-Ki-67 (#15580) was purchased from ABCAM. Fluorescein-labeled LTL (#FL-1321) and Dylight 594 anti-rabbit IgG (#DI-1594) were purchased from Vector Labs. Peroxidase marker and DAB + Substrate (#5007) were purchased from Dako.

### Generation of renal proximal tubule-specific Tsc1 gene-knockout mice

*Tsc1*-floxed mice (Strain Name: STOCK *Tsc1*^tm1Djk^/JNju. Stock Number: J005680) were purchased from Nanjing Biomedical Research Institute of Nanjing University. The *γGT-Cre* mice (Strain Name: STOCK Tg (gGT1-CRE) M3Egn/J. Stock Number: 012841) were purchased from The Jackson Laboratory. Animals were housed at the veterinary facility (West 24th building), Shanghai Medical School, Fudan University. Animal care and all experimental procedures were approved by Fudan University Basic Medical School’s Experimental Animal Ethical Committee (No. 20150119-056), and complied with the guidelines of National Institutes of Health. Renal proximal tubule cell-specific homozygous *Tsc1*-knockout (*Tsc1*^ptKO^) mice were generated by crossing *Tsc1*^*flox/flox*^ mice with a transgenic mouse line expressing Cre recombinase under the control of the gamma-glutamyl transpeptidase promoter (*γGT-Cre*)^11,35^. The *γGT-Cre* mouse line has been used to successfully delete different genes of interest selectively in the renal proximal tubule^35,36^. *Tsc1*^*flox/flox*^ littermates lacking the *γGT-Cre* transgene (*Tsc1*^*flox/flox*^*;γGT-Cre*^−^) were used as control (*Tsc1*^Ctrl^) mice, as depicted in Supplemental Fig. S1. The genotyping process was the criteria for the inclusion/exclusion (all the *Tsc1*^Ctrl^/*Tsc1*^ptKO^ mice were included and the rest were excluded), which was not described before. For each group used in experiments, at least five mice were guaranteed for the adequate power to detect a prespecified effect size. All mice were maintained and handled according to protocols approved by the NIH Animal Care and Use Committee. Block randomization was applied for the animal studies during the experiment and assessing the outcome.

### Cell culture

NRK cells were from Prof. Lu Limin (Department of Physiology, Fudan University), which was obtained from the cell bank of Chinese Academy of Sciences (China). The NRK cells were cultured in high-glucose DMEM media (Gibco) containing 10% fetal bovine serum (Gibco), maintained at 37 °C in a 5% CO_2_ cell incubator (Thermofisher).

### *Tsc1* RNA interference and metformin and dorsomorphin treatment of NRK cells

After the NRK cells were seeded at 3 × 10^5^ cells per well in 12-well plates for 24 h, *Tsc1* siRNA (25 nM) was transfected for 42 h. The siRNA sequence was 5′-CGCCTTTATGGAATGTACCCTTGTA-3′, purchased from Gene Pharma, China. The cells were then treated with 3 mM metformin (Sangon Biotech) with or without 10 μM dorsomorphin (Selleck Chemicals) for 2 h. The NRK cells were lysed with radioimmunoprecipitation assay (RIPA) buffer (Sangon Biotech) containing protease inhibitors on ice for 40 min before centrifugation at 12,500 rpm for 20 min for collection of cell lysates. Equal amounts of protein samples were loaded onto 10% sodium dodecyl sulfate polyacrylamide gel electrophoresis (SDS-PAGE) for immunoblotting analysis as we described previously^37–39^.

### PCR primers and genotyping

Genomic DNA was isolated from mouse ear or toe biopsy samples for polymerase chain reaction (PCR) genotyping. PCR primers used for the floxed *Tsc1* allele are 5′-GTCACGACCGTAGGAGAAGC-3′ and 5′-GAATCAACCCCACAGAGCAT-3′, while *γGT-Cre* transgene-detecting primers are 5′-AGGTGTAGAGAAGGCACTTAGC-3′ and 5′-CTAATCGCCATCTTCCAGCAGG-3′. PCR conditions for both floxed-*Tsc1* and *γGT-Cre* are 94 °C for 3 min followed by 94 °C for 30 s, 65 °C for 60 s, and 72 °C for 60 s for 36 cycles, with an additional 2-min extension at 72 °C.

### Histologic examination, immunohistochemistry, immunofluorescence, TUNEL stain, and immunoblotting analysis

Mouse kidneys were fixed in 4% paraformaldehyde for paraffin-embedded kidney sections (5 μm), which were then deparaffinized and rehydrated for the following staining techniques. For histologic examination, H&E staining was performed using the standard methods^39^. Immunohistochemistry and immunofluorescence staining were performed as described previously^38,39^. Briefly, rehydrated kidney sections were subjected to antigen retrieval using the Antigen Unmasking Solution purchased from Vector, followed by blocking with 5% normal goat serum. The sections were then incubated with primary antibodies (indicated in the respective figures) at 4 °C overnight, washed three times in PBS. For immunohistochemistry, the sections were subsequently incubated in a secondary antibody (peroxidase marker, Dako) at 37 °C for 1 h. Then the signals were visualized using 1:50 diluted DAB (Dako), followed by counterstaining with hematoxylin and capturing images using a Nikon microscope. For immunofluorescence staining, after incubation with the primary antibodies indicated and washing with PBS, the sections were incubated with Dylight 594-conjugated secondary antibodies and LTL for 1 h, and images were captured using the Zeiss microscope system running on the ZEN software. For terminal deoxynucleotidyl transferase dUTP nick end labeling (TUNEL) stain, sections were processed as described above for the IHC and labeled with TdT using the In-Situ Cell Death Detection Kit (Roche). Image J was applied for the calculation of the immunohistochemical-positive area. Staining was visualized using 1:50 diluted DAB (Dako). Immunoblotting analyses were performed as we described previously^37–39^.

### Measurement of kidney function

Serum creatinine, albumin, and blood urea nitrogen (BUN) levels were measured as previously described^40,41^. As for lactic acid in tissue levels, the same region of cortical tissues of mice was homogenized in RIPA cell lysis buffer, centrifuged at 12,500 rpm at 4 °C for 20 min, and the supernatant was collected as the samples. Blood samples were collected from mice at different ages indicated in the corresponding figures. Serum creatinine, albumin, and BUN levels were immediately measured according to the instruction of Creatinine, Albumin, and Blood Urea Nitrogen Reagent Set (Pointe Scientific). Urine samples were collected simultaneously from mice at different ages and monitored by SDS-PAGE and Coomassie stain.

### ELISA measurement

Culture media were collected from NRK cells treated as indicated above. The collected media were centrifuged at 2000–3000 rpm for 20 min, and the supernatants were collected for detection of IL-1β secretion according to the manufacturer’s instructions of the IL-1 beta Rat ELISA Kit (Invitrogen).

### Masson trichrome staining

Kidney sections (5 μm) were deparaffinized, rehydrated, and stained with the Masson Trichrome Stain Kit according to the manufacturer’s instructions (Sigma-Aldrich).

### Sirius red staining

The slides were incubated with a 0.1% sirius red solution dissolved in aqueous saturated picric acid for 1 h, washed in acidified water (0.5% hydrogen chloride), dehydrated, and mounted with DPX Mounting. Collagen and non-collagen components were stained red and orange, respectively.

### Statistical analysis

Data are presented as means ± SEM for at least three separate experiments (each in triplicate). An estimate of variation within each group is being statistically compared. An unpaired *t* test was used for statistical analysis; ANOVA and Bonferroni *t* tests were used for multiple group comparisons using GraphPad Prism 7. A *P* value < 0.05 (*P) compared with control was considered statistically significant.

## Results

### *Tsc1* deletion in renal proximal tubules caused striking kidney growth

*Tsc1* gene-floxed mice (*Tsc1*^*flox/flox*^) were crossed with transgenic mice expressing the Cre recombinase under the control of the gamma-glutamyl transpeptidase (*γGT*) promoter to generate a proximal tubule-specific *Tsc1* gene-knockout (*Tsc1*^ptKO^) mouse line. Sex-matched littermates with a genotype of *Tsc1*^*flox/flox*^*;γGT-Cre*^*−*^ were used as control (called *Tsc1*^Ctrl^ mice hereafter) (Fig. S1a). *Tsc1*^*flox/flox*^ mice were identified by PCR as those carrying only the *Tsc1*-floxed allele (230 bp), while *Tsc1*^*flox/wt*^ mice were those carrying both the *Tsc1*-floxed allele (230 bp) and a wild-type allele (192 bp) of the *Tsc1* gene. The *γGT-Cre* gene was detected by PCR as a 370-bp band in *Tsc1*^*ptKO*^ mice but not in *Tsc1*^*Ctrl*^ mice (Fig. S1b). By 4 weeks of age, *Tsc1*^ptKO^ mice exhibited a smaller body size (Fig. S1c), but had strikingly larger kidneys (Fig. S1d), compared with *Tsc1*^Ctrl^ littermates.

**Fig. 1:**
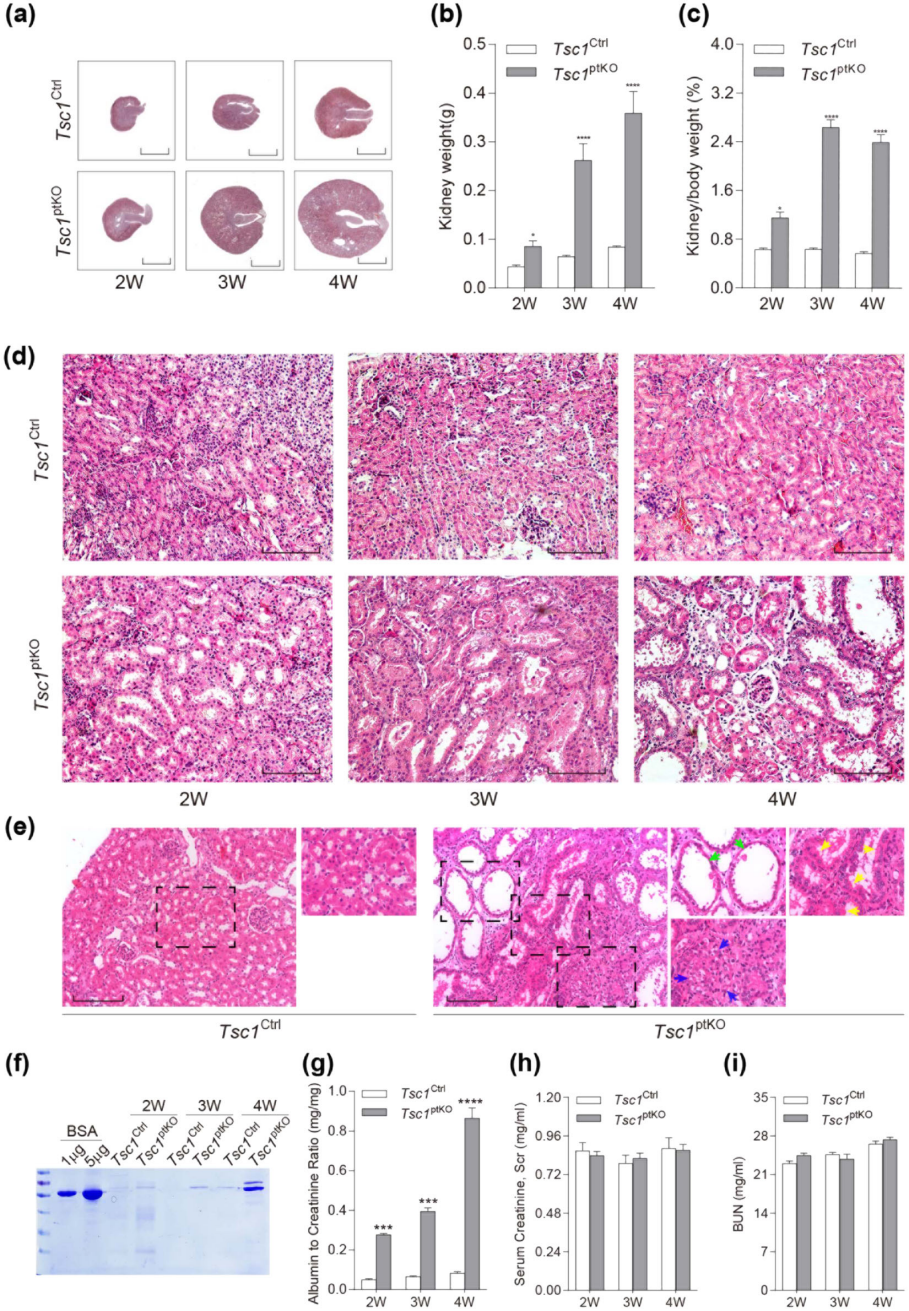
Characterization and renal function of renal proximal tubule cell-specific *Tsc1*-knockout (*Tsc1*^ptKO^) mice. **a** Representative whole kidney sections with H&E staining from *Tsc1*^ptKO^ and *Tsc1*^Ctrl^ mice at 2–4 weeks of age, with *Tsc1*^ptKO^ mice showing increased kidney size. *Tsc1*^ptKO^ mice (*n* = 7) had increased **b** kidney weights and **c** kidney-to-body weight ratios, compared with *Tsc1*^Ctrl^ littermates (*n* = 8). Data were from 2 to 4 weeks of age and expressed as means ± SEM, *P < 0.05, ****P < 0.0001. **d** Higher- magnification light microscopy revealed enlarged renal proximal tubules and renal cysts in *Tsc1*^ptKO^ mice, compared with *Tsc1*^Ctrl^ mice by 2–4 weeks of age. **e** Representative images were selected from both *Tsc1*^ptKO^ and *Tsc1*^Ctrl^ mice at 4 weeks of age. In detail, *Tsc1*^ptKO^ mice showed dilated renal cysts (green arrow), abnormally proliferative and hypertrophic renal tubule epithelial cells (yellow arrows), and occasional tumor-like structures (blue arrows). (Scale bar: 2 mm in all images of (**a**); 100 μm in all images of (**d**, **e**)). **f** The coomassie staining showed that the albuminuria level of *Tsc1*^ptKO^ mice was higher than that of *Tsc1*^Ctrl^ mice at 2–4 weeks, with an obvious elevation at 4 weeks of age. Data were shown of three independent experiments. **g** ACR was increased in *Tsc1*^ptKO^ mice by 2–4 weeks of age, with an obvious elevation by 4 weeks of age. Such tendency was not observed in (**h**) Scr and (**i**) BUN. Data were from 2 to 4 weeks of age mice (*n* = 7–8 each per group) and expressed as means ± SEM, ****P < 0.0001.

As shown in Fig. 1, compared with *Tsc1*^Ctrl^ littermates, *Tsc1*^ptKO^ mice developed markedly enlarged kidneys by 2 weeks of age, which continued to enlarge aberrantly by 3 and 4 weeks of age (Fig. 1a), with significant increases in kidney weight (Fig. 1b) and kidney-to-body weight ratios (Fig. 1c). Renal histology revealed hypertrophy of renal tubules in the kidneys of *Tsc1*^ptKO^ mice by 2 weeks of age, and some enlarged renal tubules became dilated after 3 weeks of age (Fig. 1d). By 4 weeks of age, *Tsc1*^ptKO^ mice developed some microscopic renal cysts (Fig. 1d, e). More detailed examination of the renal pathology under higher magnification revealed that the large kidney phenotype of *Tsc1*^ptKO^ mice was caused by both cellular hypertrophy and proliferation, leading to enlargement of renal tubules with occasional microscopic hamartoma formation (Fig. 1e).

### *Tsc1* deletion in renal proximal tubules caused moderate albuminuria

**Fig. 2:**
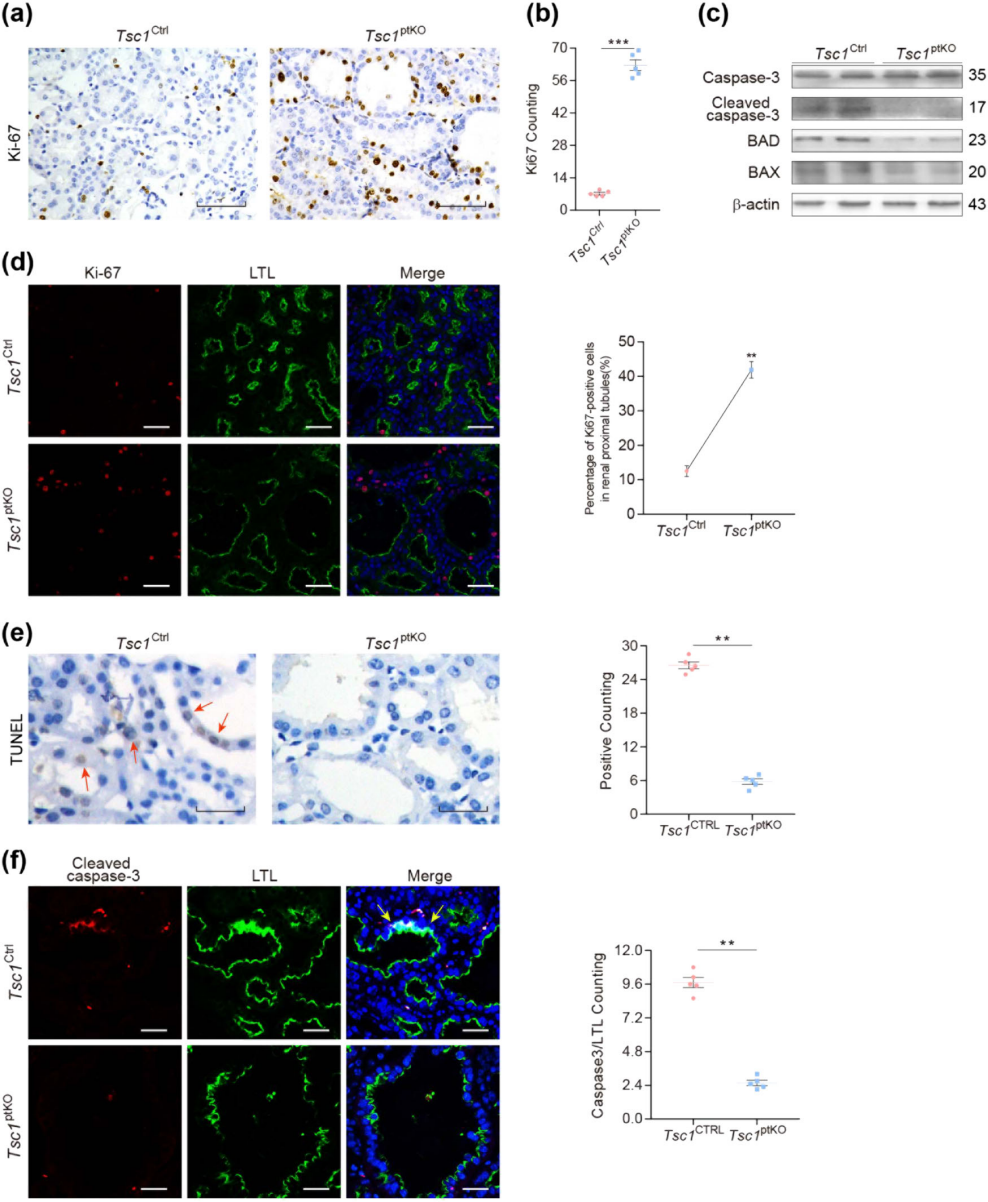
Renal proximal tubule cell-specific *Tsc1* knockout induced abnormal cellular proliferation and inhibited apoptosis by 4 weeks of age. **a** Immunohistochemistry indicated that the positive number of Ki-67 proximal tubule cells in *Tsc1*^ptKO^ mice was higher than that in *Tsc1*^Ctrl^ mice (*n* = 7). **b** The number of Ki-67-positive cells was counted in 25 random captured images for each group (5 images per mouse, 5 mice per group) at the ×400 magnification images, which indicated that the average expression of Ki-67 was higher in *Tsc1*^ptKO^ mice than *Tsc1*^Ctrl^ mice. **c** Immunoblotting showed that cleaved caspase-3, BAD, and BAX were decreased in the renal cortex of *Tsc1*^ptKO^ mice, compared with *Tsc1*^Ctrl^ mice. Data were shown of three independent experiments. **d** Double immunofluorescence of Ki-67 (red) and LTL (green) showed an increase in co-localization of Ki-67 and LTL in *Tsc1*^ptKO^ mice, compared with *Tsc1*^Ctrl^ mice. The percentage of Ki-67-positive cells was calculated from 25 randomly captured images for each group (5 images per mouse, 5 mice per group) at the original magnification of ×400 from the LTL-positive region. Only Ki-67-positive nuclei within LTL-positive tubules were counted for the numerator, which was divided by the sum of Ki-67- and DAPI-positive nuclei within the LTL-positive tubules and then multiplied by 100%. **e** TUNEL signal (red arrows) was positive in the renal tubule epithelial cells of *Tsc1*^Ctrl^ mice by TUNEL staining, but negative in *Tsc1*^ptKO^ mice. The number of TUNEL-positive cells was counted in 25 random captured images for each group (5 images per mouse, 5 mice per group) of ×400 magnification images. **f** Double immunofluorescence of cleaved caspase-3 (red) and LTL (green) showed a reduction of cleaved caspase-3 and LTL co-localization in *Tsc1*^ptKO^ mice, compared with *Tsc1*^Ctrl^ mice. The percentage of cleaved caspase-3-positive cells was calculated from 25 randomly captured images for each group (5 images per mouse, 5 mice per group) at the original magnification of ×400 from the LTL-positive region. Only Ki-67-positive nuclei within LTL-positive tubules were counted. Data were from 4 weeks of mice (*n* = 5 each per group) and expressed as means ± SEM, **P < 0.01, ***P < 0.001. (Scale bar: 50 μm in all images of (**a**), 25 μm in all images of (**e**), and 40 μm in all images of (**d**, **f**)).

To determine whether the structural damage of the kidneys shown in Fig. 1 has caused functional impairment of the kidneys in *Tsc1*^ptKO^ mice, we measured urinary protein excretion, serum creatinine, and BUN levels. As shown in Fig. 2a, urinalysis by SDS-PAGE revealed that *Tsc1*^ptKO^ mice developed mild albuminuria by 2 weeks of age that became markedly more apparent by 4 weeks of age (Fig. 1f). The proteinuria was confirmed by statistically significant increases in urinary albumin-to-creatinine ratios (Fig. 1g). The increased excretion of albumin in the urine may be explained by the data shown in Fig. 2, indicating that the renal proximal tubule cells in *Tsc1*^ptKO^ mice were activated to undergo dedifferentiation and proliferation, so their normal function of reabsorbing albumin from the glomerular filtrate might have been compromised. However, *Tsc1*^ptKO^ mice had normal serum creatinine levels (Fig. 2h) and BUN levels (Fig. 2i), indicating that the excretion of serum creatinine and BUN was not decreased.

### *Tsc1* deletion in renal proximal tubules induced cell proliferation, suppressed apoptosis, and promoted inflammation and fibrosis

Immunohistochemistry with an antibody specific for Ki-67, a marker of proliferating cells, revealed aberrant proliferation of renal tubular epithelial cells in *Tsc1*^ptKO^ mice (Fig. 2a), which was confirmed by quantitation of Ki-67-positive cells in the kidney sections of *Tsc1*^ptKO^ mice, compared with that of *Tsc1*^Ctrl^ littermates (Fig. 2b). Double-immunofluorescence staining with *Lotus Tetragonolobus Lectin* (LTL, a marker specific for proximal tubules) and the Ki-67 antibody indicated that increased proliferating cells are largely in the proximal tubules of *Tsc1*^ptKO^ mice; the increases in Ki-67-positive cells lining the renal proximal tubules were demonstrated to be statistically significant (Fig. 2d). Furthermore, immunoblotting revealed downregulation of cleaved caspase-3, Bcl-2-associated X protein (BAX), and Bcl-2-associated death promoter (BAD) in *Tsc1*^ptKO^ mice (Fig. 2c), leading to decreased apoptosis in the kidneys of *Tsc1*^ptKO^ mice, compared with that of *Tsc1*^Ctrl^ mice, as indicated by TUNEL assays and quantitative data (Fig. 2e). The decreased apoptosis was consistent with decreases in cleaved caspase-3-positive cells in *Tsc1*^ptKO^ mice, indicated by immunofluorescence staining of kidney sections with LTL and an antibody specific for cleaved caspase-3 and the corresponding quantitative data (Fig. 2f).

**Fig. 3:**
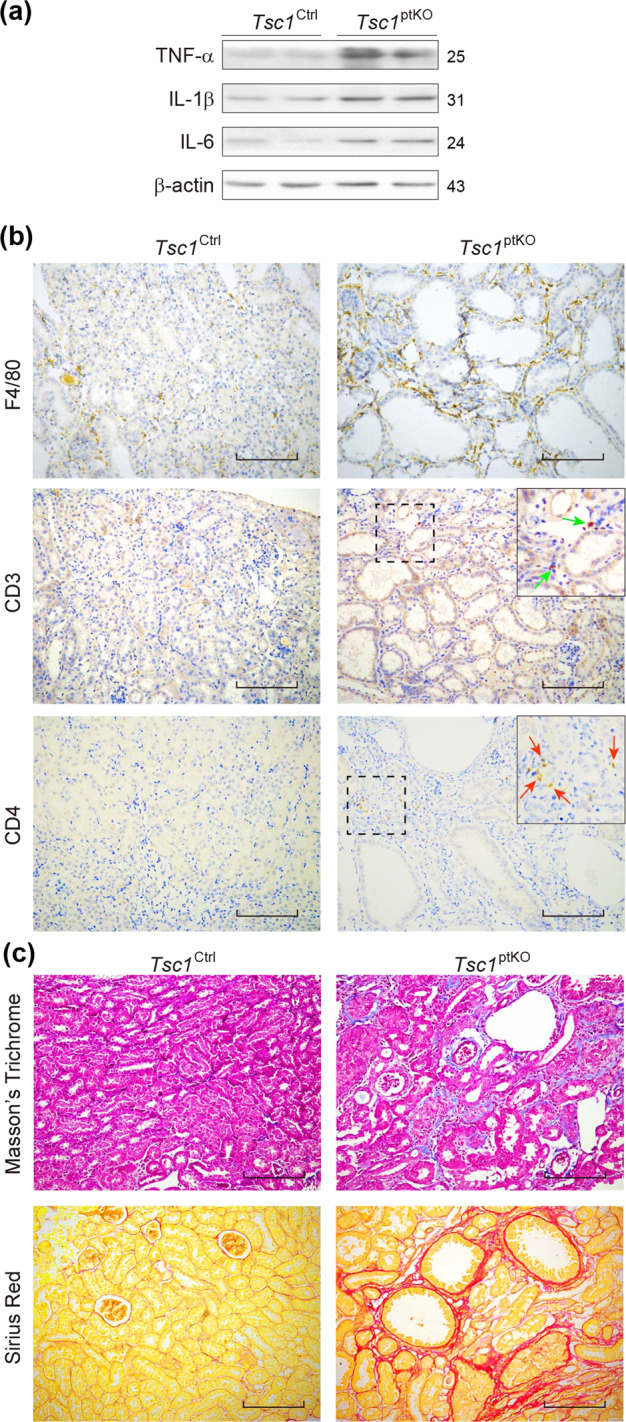
Renal proximal tubule cell-specific *Tsc1* knockout promoted interstitial inflammation as well as fibrosis by 4 weeks of age. **a** Immunoblotting showed that the expression of TNF-α, IL-1β, and IL-6 in the renal cortex of *Tsc1*^ptKO^ mice was higher than that of *Tsc1*^Ctrl^ mice. Data were shown of three independent experiments. **b** Immunohistochemistry showed that the renal interstitial F4/80, as well as CD3- (green arrow) and CD4- (red arrows) positive staining cells were increased in *Tsc1*^ptKO^ mice, compared with *Tsc1*^Ctrl^ mice. **c** More renal interstitial collagen formation was observed in *Tsc1*^ptKO^ mice by Masson’s trichrome and sirius red staining, compared with *Tsc1*^Ctrl^ mice. Data were from 4 weeks of age mice (*n* = 7–8 each per group) (scale bar: 100 μm in all images of (**b**, **c**)).

In addition, immunoblotting analysis of renal cortical homogenates unveiled increases in the expression levels of TNF-α, IL-1β, and IL-6 in *Tsc1*^ptKO^ mice, compared with those in *Tsc1*^Ctrl^ mice (Fig. 3a). These findings may be explained by increases in inflammatory cell infiltrates, including F4/80-positive macrophages as well as CD3- or CD4-positive lymphocytes, in the interstitial areas of *Tsc1*^ptKO^ mice, compared with those of *Tsc1*^Ctrl^ mice, revealed by immunohistochemical staining with the indicated antibodies (Fig. 3b). Moreover, Masson’s trichrome and sirius red staining revealed renal interstitial fibrosis in *Tsc1*^ptKO^ mice (Fig. 3c).

### Daily metformin treatment reduced aberrant kidney growth and attenuated albuminuria in *Tsc1*^ptKO^ mice

**Fig. 4:**
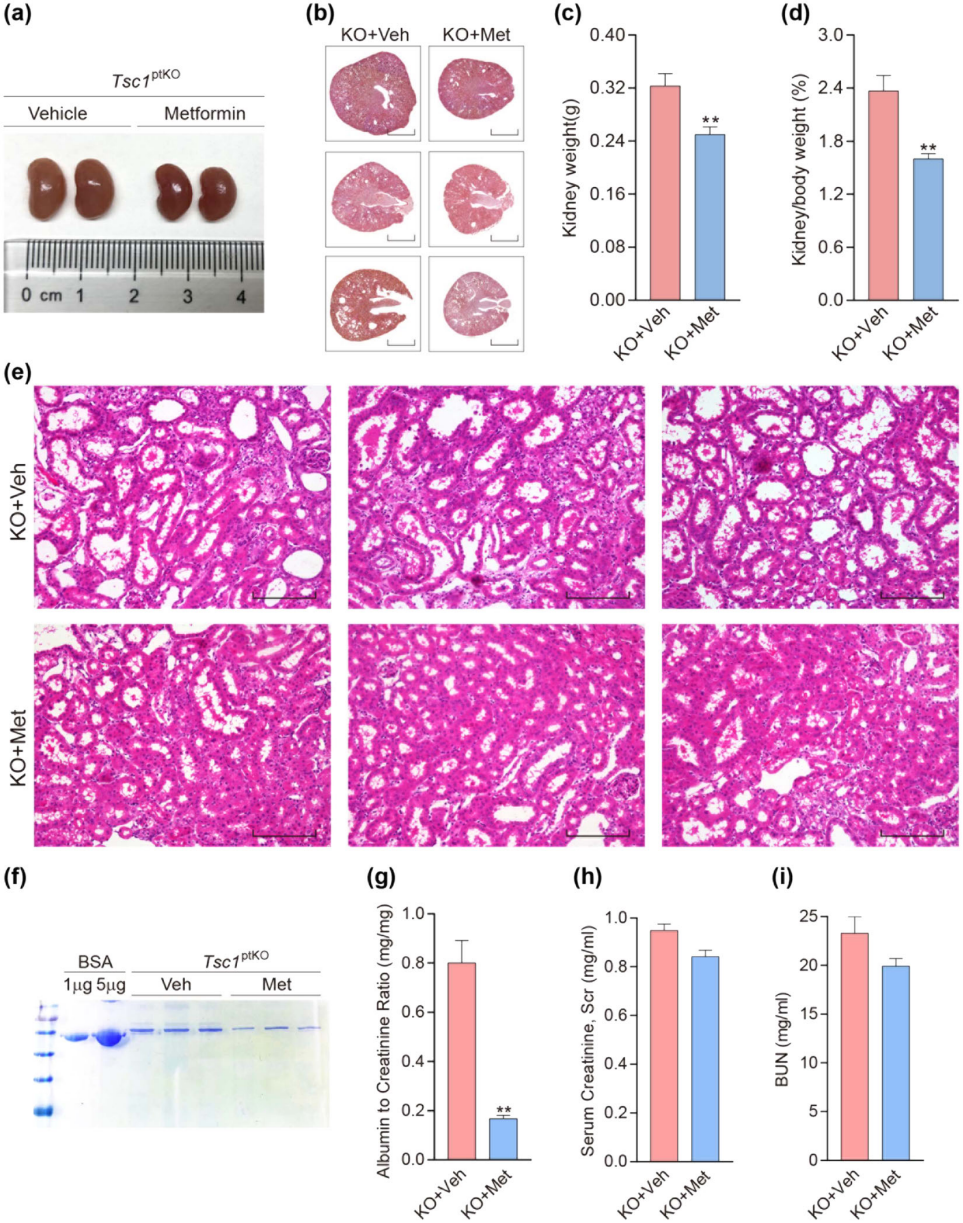
Metformin prevented *Tsc1* deletion-induced kidney growth and ameliorated *Tsc1* deletion-induced renal injuries by 4 weeks of age. Representative **a** kidney images and **b** kidney sections of metformin (20 mg/mL in vehicle, i.p.) and vehicle- (0.9% saline, i.p.) treated *Tsc1*^ptKO^ mice. The metformin- and vehicle-treated mice were given daily weight-adjusted i.p. injections from 1 to 4 weeks of age. Metformin prevented **c** kidney weights and **d** kidney- to-body weight ratios in *Tsc1*^ptKO^ mice, compared with vehicle-treated group. Data were from 4 weeks of age mice (*n* = 5–6 each per group) and expressed as means ± SEM, **P < 0.01. **e** Higher-magnification light microscopy demonstrated that metformin decreased renal cysts, as well as suppressed enlarged proximal tubules in *Tsc1*^ptKO^ mice, compared with vehicle-treated group. (Scale bar: 2 mm in all images of (**b**); 100 μm in all images of (**e**)). **f** Coomassie staining showed that metformin reduced albuminuria in *Tsc1*^ptKO^ mice, compared with vehicle-treated group. Data were shown of three independent experiments. Metformin significantly decreased **g** ACR in *Tsc1*^ptKO^ mice, compared with vehicle-treated group. Besides, reductions of **h** Scr and **i** BUN were detected in *Tsc1*^ptKO^ mice, although there was no statistical significance. Data were from 4 weeks of age mice (*n* = 5–6 each per group) and expressed as means ± SEM, **P < 0.01.

Previous studies have shown that metformin inhibits renal fibrosis in a mouse model of UUO^27,28^ and attenuates renal injury in PKD^21^. To test the effect of metformin on *Tsc1* deletion-induced abnormal kidney growth, we treated *Tsc1*^ptKO^ mice with metformin by intraperitoneal injection once a day starting from 1 week of age for 3 consecutive weeks. Compared with vehicle-treated *Tsc1*^ptKO^ mice, metformin treatment markedly reduced kidney size (Fig. 4a, b), kidney weight (Fig. 4c), and kidney/body weight ratios (Fig. 4d) in *Tsc1*^ptKO^ mice. There was a 22.7% reduction in kidney weight and a 32.2% reduction in kidney-to-body weight ratios (Fig. 4c, d). Compared with the vehicle-treated group, metformin treatment also markedly attenuated cystic renal lesions (Fig. 4b, e). Importantly, metformin treatment effectively preserved renal function, evidenced by marked decreases in proteinuria (Fig. 4f) and urinary albumin-to-creatinine ratios (Fig. 4g), with no significant alterations in the normal serum creatinine (Fig. 4h) and BUN levels in *Tsc1*^ptKO^ mice (Fig. 4i), compared with vehicle-treated *Tsc1*^ptKO^ mice. Thus, metformin effectively reduced the abnormal enlargement of kidneys and mitigated the albuminuria in *Tsc1*^ptKO^ mice.

### Metformin treatment inhibited excessive proliferation, induced apoptosis, suppressed inflammation, and attenuated fibrosis in the kidneys of *Tsc1*^ptKO^ mice

**Fig. 5:**
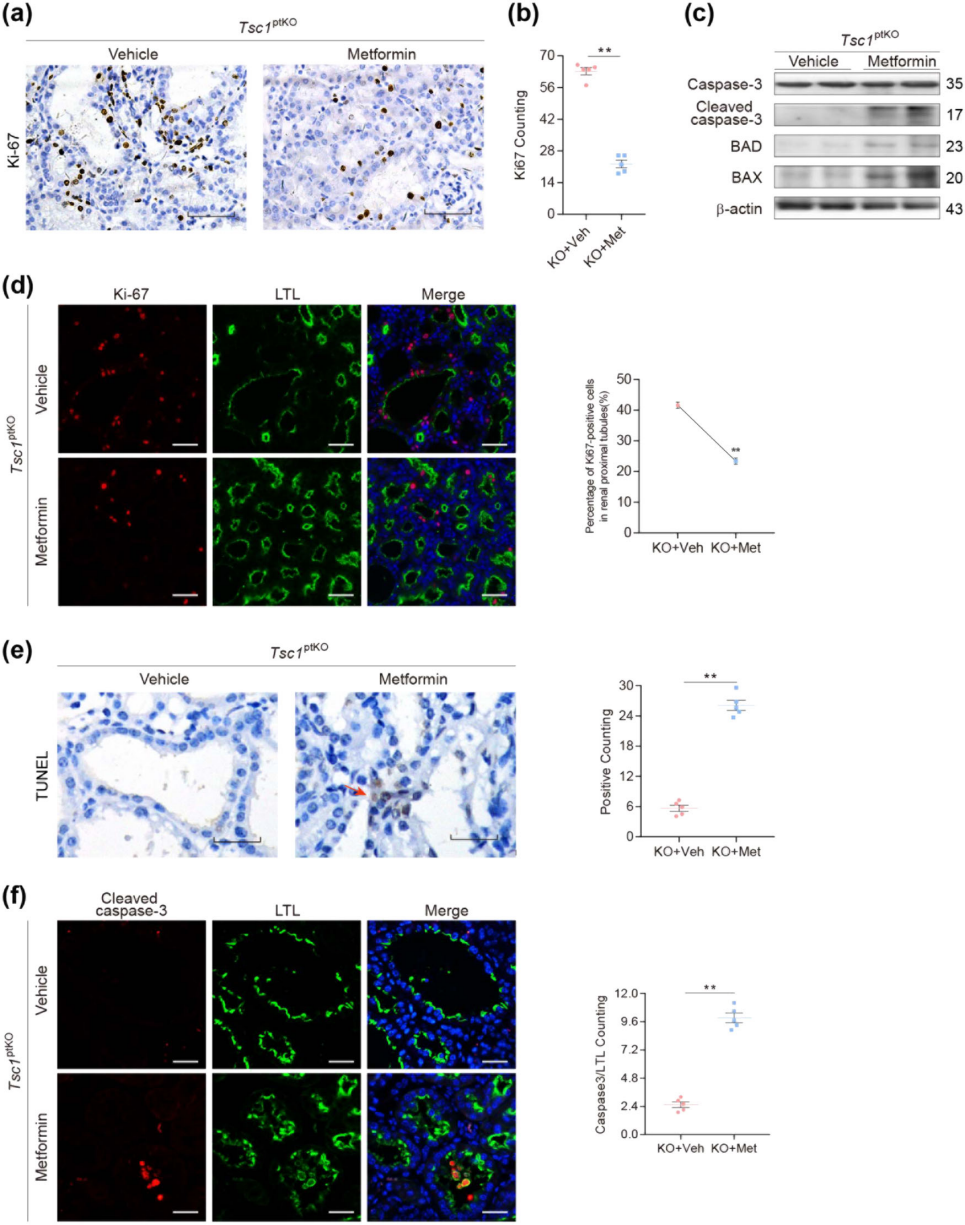
Metformin reduced proximal tubule cell proliferation and induced apoptosis in *Tsc1*^ptKO^ mice by 4 weeks of age. **a** Ki-67 staining by immunohistochemistry indicated that metformin decreased the number of Ki-67-positive proximal tubule cells expressed in *Tsc1*^ptKO^ mice, compared with vehicle-treated group. **b** The number of Ki-67-positive cells was counted in 25 random captured images for each group (5 images per mouse, 5 mice per group) at the ×400 magnification images, which demonstrated that metformin reduced the average expression of Ki-67 in *Tsc1*^ptKO^ mice, compared with vehicle-treated group. **c** Immunoblotting showed that metformin upregulated cleaved caspase-3, BAD, and BAX in the renal cortex of *Tsc1*^ptKO^ mice, compared with the vehicle-treated group. Data were shown of three independent experiments. **d** Double immunofluorescence of Ki-67 (red) and LTL (green) showed a decrease of Ki-67 and LTL co-localization in metformin-treated *Tsc1*^ptKO^ mice, compared with vehicle-treated group. The percentage of Ki-67-positive cells was calculated from 25 randomly captured images for each group (5 images per mouse, 5 mice per group) at the original magnification of ×400 from the LTL-positive region. Only Ki-67-positive nuclei within LTL-positive tubules were counted for the numerator, which was divided by the sum of Ki-67- and DAPI-positive nuclei within the LTL-positive tubules and then multiplied by 100%. **e** Reappearance of TUNEL-positive signals (red arrows) was demonstrated in the renal tubule epithelial cells of metformin-treated *Tsc1*^ptKO^ mice, which were barely traced in vehicle-treated group. The number of TUNEL-positive cells was counted in 25 random captured images for each group (5 images per mouse, 10 mice per group) of ×400 magnification images. **f** Double immunofluorescence of cleaved caspase-3 (red) and LTL (green) showed that metformin promoted the co-localization of cleaved caspase-3 and LTL in *Tsc1*^ptKO^ mice, compared with the vehicle-treated group. The percentage of cleaved caspase-3-positive cells was calculated from 50 randomly captured images for each group (5 images per mouse, 5 mice per group) at the original magnification of ×400 from the LTL-positive region. Only Ki-67-positive nuclei within LTL-positive tubules were counted. Data were from 4 weeks of age mice (*n* = 5 each per group) and expressed as means ± SEM, **P < 0.01 (scale bar: 50 μm in all images of (**a**), 25 μm in all images of (**e**), and 40 μm in all images of (**d**, **f**)).

Previous studies have demonstrated that metformin inhibits the survival and growth of cancer cells^29,30^. To further investigate the beneficial effects of metformin in our *Tsc1*-knockout mouse model, we analyzed cell proliferation, apoptosis, interstitial inflammation, and fibrosis in the kidneys of *Tsc1*^ptKO^ mice treated with metformin, compared with vehicle-treated *Tsc1*^ptKO^ mice. As shown in Fig. 5a, b, immunohistochemical staining with antibodies against Ki-67, a marker for proliferating cells, revealed a significant reduction in Ki-67-positive cells in the kidneys of *Tsc1*^ptKO^ mice. Double-immunofluorescence staining with LTL (Green) and Ki-67 (Red) with statistical analysis demonstrated that the proliferating cells were primarily among the epithelial cells lining renal proximal tubules and renal cysts in *Tsc1*^ptKO^ mice, and were markedly reduced by treatment with metformin (Fig. 5d).

In addition, we observed marked upregulation of several apoptosis mediators, including cleaved caspase-3, BAD, and BAX in metformin-treated *Tsc1*^ptKO^ mice, compared with vehicle-treated *Tsc1*^ptKO^ mice (Fig. 5c). Such molecular signaling alterations were accompanied by increases in TUNEL-positive cells in metformin-treated *Tsc1*^ptKO^ mice relative to vehicle-treated *Tsc1*^ptKO^ mice, which were also indicated in the statistical analysis of TUNEL-positive cells (Fig. 5e). Furthermore, double-immunofluorescence staining with LTL (green) and cleaved caspase-3 and the corresponding quantitative data confirmed that metformin treatment induced marked apoptosis of the LTL-positive renal proximal tubular epithelial cells, with some apoptotic cells having sloughed off the basement membrane and fragmented within the lumen, resulting in shrunken renal cysts (Fig. 5f). These results suggest that metformin has both antiproliferative and proapoptotic effects on the renal proximal tubular epithelial cells in *Tsc1*^ptKO^ mice.

**Fig. 6:**
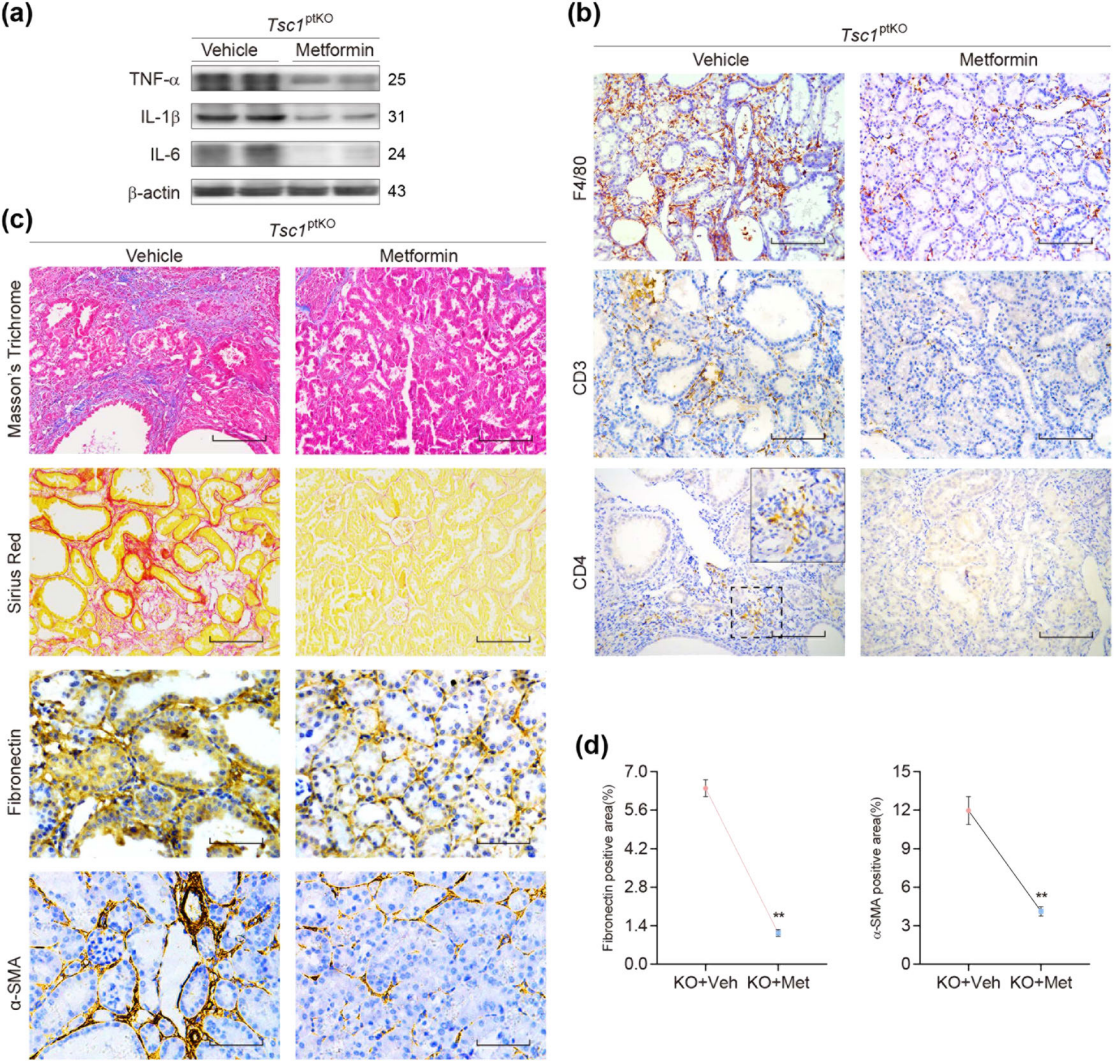
Metformin suppressed the interstitial inflammation as well as fibrosis in *Tsc1*^ptKO^ mice by 4 weeks of age. **a** Immunoblotting showed that metformin reduced the expression of TNF-α, IL-1β, and IL-6 in the renal cortex of *Tsc1*^ptKO^ mice, compared with the vehicle-treated group. Data were shown of three independent experiments. **b** Immunohistochemistry showed that metformin decreased renal interstitial F4/80, as well as reductions of CD3- and CD4-positive staining cells in *Tsc1*^ptKO^ mice, compared with the vehicle-treated group. **c** Decreased renal interstitial collagen formation was indicated by Masson’s trichrome and sirius red staining after metformin treatment in *Tsc1*^ptKO^ mice, compared with the vehicle-treated group. In the meantime, decreased renal interstitial fibronectin and α-SMA were demonstrated by immunohistochemistry in metformin-treated *Tsc1*^ptKO^ mice, compared with the vehicle-treated group. **d** The drop of fibronectin and α-SMA-positive areas was observed after the treatment of metformin in *Tsc1*^ptKO^ mice, compared with the vehicle-treated group. Only fibronectin and α-SMA-positive areas were analyzed for the numerator, which was divided by the total area of the ×400 magnification images and then multiplied by 100%. Data were from 4 weeks of age mice (*n* = 5 each per group) (scale bar: 100 μm in all images of (**b**), upper four images of (**c**), and 50 μm in lower four images of (**c**)).

When we performed additional experiments to compare metformin-treated *Tsc1*^ptKO^ mice with vehicle-treated *Tsc1*^ptKO^ mice, we found that metformin markedly decreased the levels of TNF-α, IL-1β, and IL-6, as unveiled by immunoblotting (Fig. 6a). Metformin also markedly decreased F4/80-, CD3-, or CD4-positive inflammatory cells, revealed by immunohistochemistry (Fig. 6b). Moreover, Masson’s Trichrome and sirius red staining detected a marked reduction in collagen deposition, while immunohistochemistry revealed a marked reduction in α-SMA-positive myofibroblasts and fibronectin deposition in *Tsc1*^ptKO^ mice in response to metformin treatment (Fig. 6c), which were also indicated by the quantitative data of fibronectin- and α-SMA-positive areas (Fig. 6d). These results suggest that in addition to an anti-inflammatory effect, metformin also has an anti-fibrotic effect in treating *Tsc1*^ptKO^ mice.

### The efficacy of metformin in treating cystic kidney disease was largely mediated by upregulation of AMPK phosphorylation

**Fig. 7:**
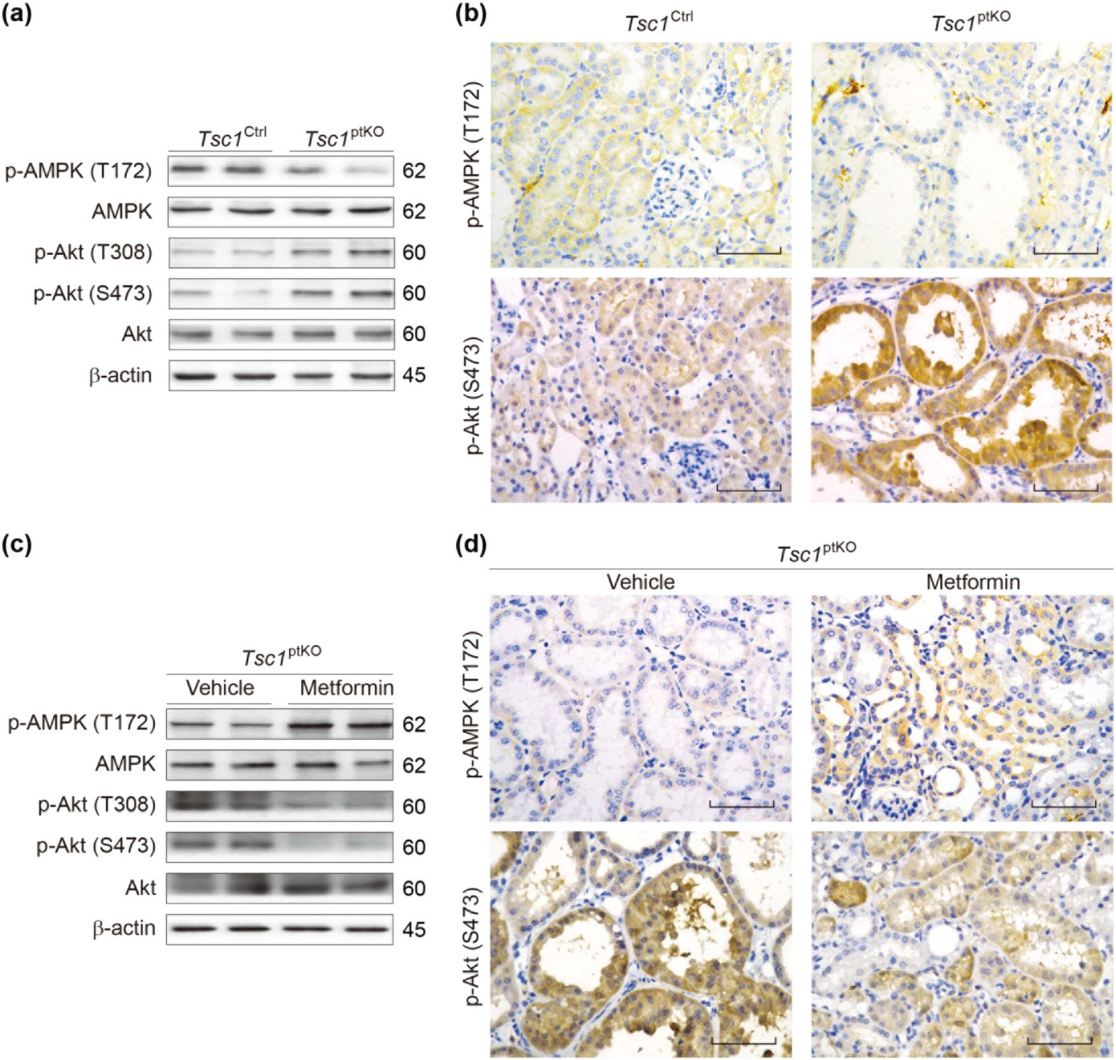
Metformin restored AMPK phosphorylation, whereas suppressed Akt phosphorylation in *Tsc1*^ptKO^ mice kidney by 4 weeks of age. **a** Immunoblotting showed that p-AMPK (Thr-172) was decreased, whereas p-Akt (Ser-473) and (Thr-308) were increased in the renal cortex of *Tsc1*^ptKO^ mice, compared with *Tsc1*^Ctrl^ mice. **b** Immunohistochemistry showed a reduction of p-AMPK (Thr-172), whereas an upregulation of p-Akt (Ser-473) was observed in proximal tubule cells of *Tsc1*^ptKO^ mice. Data were from 4 weeks of age mice (*n* = 5 each per group). **c** Immunoblotting indicated that metformin upregulated p-AMPK (Thr-172), whereas suppressed p-Akt (Ser-473) and (Thr-308) in the renal cortex of *Tsc1*^ptKO^ mice, compared with the vehicle-treated group. **d** Immunohistochemistry demonstrated that metformin restored p-AMPK (Thr-172), but suppressed p-Akt (Ser-473) in the proximal tubule epithelial cells of *Tsc1*^ptKO^ mice, compared with vehicle-treated group. Data were from 4 weeks of age mice (*n* = 5 each per group). Immunoblotting data were shown of three independent experiments (scale bar: 100 μm in all images of (**b**, **d**)).

Previous studies have demonstrated that Akt mediates the survival of renal proximal tubular cells because apoptosis induced by serum withdrawal, hydrogen peroxide, etoposide, or excess free arachidonic acid is inhibited by activation of Akt^42^. Renal proximal tubular cells make up the bulk of the renal cortex. Interestingly, our additional mechanistic studies by immunoblotting of renal cortical homogenates indicated that *Tsc1*^ptKO^ mice exhibited discernible decreases in Thr-172-phosphorylated AMPK along with parallel increases in Ser-473- and Thr-308-phosphorylated Akt levels, compared with *Tsc1*^Ctrl^ mice (Fig. 7a). Immunohistochemical staining with antibodies specific for phospho-AMPK and phosphor-Akt, respectively, confirmed that such signaling alterations were largely localized to the epithelial cells lining the activated proximal tubules and renal cysts in *Tsc1*^ptKO^ mice (Fig. 7b). Importantly, compared with vehicle-treated *Tsc1*^ptKO^ mice, metformin-treated *Tsc1*^ptKO^ mice showed upregulated AMPK phosphorylation and inhibited Akt phosphorylation, revealed by immunoblotting (Fig. 7c) and confirmed by immunohistochemistry (Fig. 7d).

**Fig. 8:**
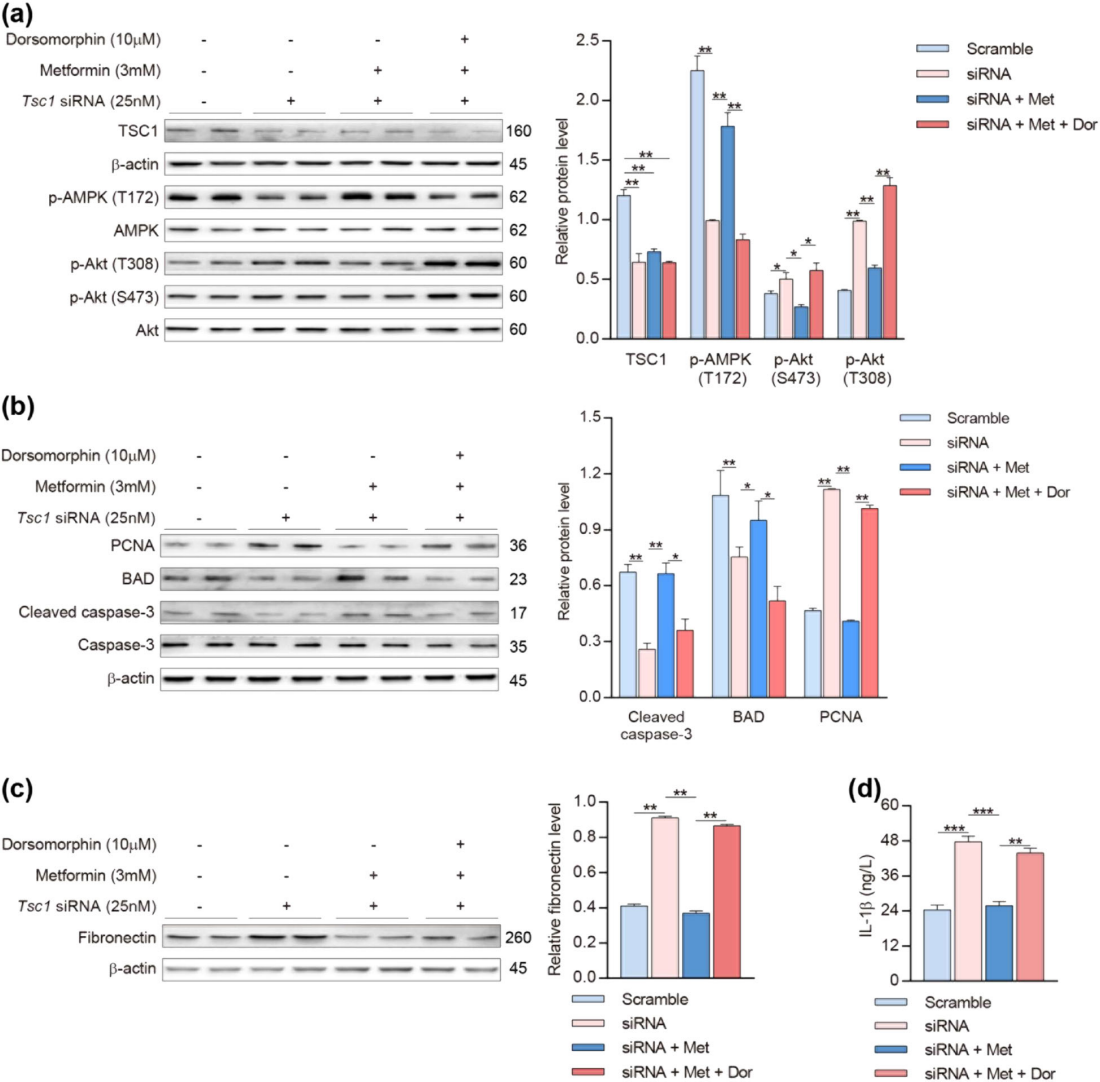
Metformin performed the effects of anti-proliferation, pro-apoptosis, and suppressing inflammation or fibrosis through AMPK phosphorylation. *Tsc1* siRNA (25 nM) was transfected for 48 h, during which metformin (3 mM) was added 6 h and dorsomorphin (10 µM) was added 2 h before transfection termination in NRK cells. **a** Metformin reversed the downregulation of p-AMPK (Thr-172) and the upregulation of p-Akt (Ser-473) and (Thr-308) in *Tsc1*- knockdown NRK cells (Lines 5 and 6), whereas neutralized under the stimulation of dorsomorphin, which reduced p-AMPK (Thr-172) but raised p-Akt (Ser-473) and (Thr-308) again (Lines 7 and 8). **b** Metformin decreased the expression of PCNA, whereas promoted cleaved caspase-3, BAD in *Tsc1*-knockdown NRK cells (Lines 5 and 6); however, stimulation of dorsomorphin revived the expression of PCNA, while reduced cleaved caspase-3, BAD, and BAX (Lines 7 and 8). **c** Metformin reversed the high expression of fibronectin in *Tsc1*-knockdown NRK cells (Lines 5 and 6), whereas dorsomorphin upregulated fibronectin again under the treatment of metformin (Lines 7 and 8). **d** IL-1β in the supernatant of the NRK cells was determined by ELISA, which demonstrated that metformin suppressed the aberrantly increased IL-1β secretion after *Tsc1* knockdown, while the stimulation of dorsomorphin re-increased the secretion of IL-1β. Data were shown of three independent experiments and expressed as means ± SEM, *P < 0.05, **P < 0.01, ***P < 0.001.

The in vivo animal data demonstrated above suggest that metformin treatment effectively reduces *Tsc1* deletion-induced aberrant kidney growth by upregulating AMPK phosphorylation, leading to inhibition of the pro-survival factor, Akt, and activation of the proapoptotic factors, BAD and cleaved caspase-3, in the renal proximal tubular cells. To confirm such a molecular mechanism, we performed additional experiments in cultured NRK cells, a rat renal proximal tubular cell line^43^. As shown in Fig. 8a, siRNA-induced knockdown of Tsc1 downregulated Thr-172-phosphorylated AMPK, but upregulated Ser-473- and Thr-308-phosphorylated Akt. Such effects were prevented by metformin treatment. These effects were reversed by treatment with dorsomorphin, an AMPK inhibitor.

Moreover, the knockdown of *Tsc1* also elevated PCNA and reduced the expression of cleaved caspase-3 and BAD. Metformin treatment reduced PCNA, but increased cleaved caspase-3 and BAD; these effects were antagonized by treatment with the AMPK inhibitor, dorsomorphin (Fig. 8b). Our further experiments indicated that the knockdown of *Tsc1* upregulated fibronectin and IL-1β. These changes were reversed by metformin treatment, but re-upregulated by dorsomorphin (Fig. 8c, d). Thus, our in vitro cell culture studies have confirmed our in vivo animal data, indicating that the restored phosphorylation level of AMPK mediates the pharmacological effect of metformin in suppressing cell proliferation, promoting apoptosis, and inhibiting the production of inflammatory cytokines as well as fibrotic factors.

## Discussion

AMPK inactivation has been observed in multiple diseases, such as enlarged cysts in PKD^28^, uncontrolled proliferation in breast cancer^23^, renal inflammation induced by cyclophosphomide^44^, and lung fibrosis after bleomycin stimulation^34^. In our study, downregulated AMPK phosphorylation was also observed in the enlarged kidneys of *Tsc1*^ptKO^ mice, which also exhibited aberrant proliferation of proximal tubule cells, renal interstitial inflammation, and fibrosis. The reduced AMPK phosphorylation in TSC is presumably caused by decreased AMP-to-ATP ratio^19^. However, the specific mechanisms regulating AMPK phosphorylation levels still need to be further elaborated under the influence of metformin.

Our results indicate that metformin treatment inhibits cell proliferation and induces apoptosis in the renal proximal tubules of *Tsc1*^ptKO^ mice by upregulating AMPK phosphorylation, in the meantime downregulating Akt phosphorylation. Our findings support the notion that AMPK phosphorylation-dependent inactivation of Akt inhibits cell proliferation and promotes apoptosis. Previous studies demonstrated that AMPK halts cycling cells in G1 phase by activating phosphorylation of p53 as well as inhibiting cycling D1, leading to cell-cycle arrest and exerting anticancer effect^23^. Moreover, as a critical cycling procedure, mitosis is also under the regulation of AMPK by phosphorylating PPP1R12C at Ser452, which is elevated in mitotic cells^19^. All these researches illustrate the critical role of AMPK in regulating proliferation and apoptosis, which inspires a novel idea applying AMPK activator in dealing with tumor cells.

Therefore, as potent AMPK activators, either resveratrol or metformin could slow the progress of cancers in ovary^25^ or breast^23^ through inhibiting cell proliferation and promoting apoptosis by activating AMPK phosphorylation^45^, which demonstrates the efficacy of AMPK against tumorigenesis. Of note, due to the mutual phosphorylation regulation of AMPK and Akt, the inactivation of AMPK prominently facilitates the Akt phosphorylation^46^, which downregulates proapoptotic factors such as BAX and BAD, leading to inhibition of cell apoptosis^47^. In our study, metformin-treated *Tsc1*^ptKO^ mice showed an evident proapoptotic effect by TUNEL assay, accompanied with immunoblotting showing downregulation of cleaved caspase-3, BAX, and BAD, as well as reduction of Ki-67. In vitro cell culture studies indicated that the effect of metformin in regulating proliferation and apoptosis was reversed by dorsomorphin, which further demonstrated the involvement of AMPK phosphorylation after metformin performance. Such a cellular mechanism may explain why metformin reduced renal cystogenesis and suppressed aberrant enlargement of the kidneys in *Tsc1*^ptKO^ mice.

Apart from uncontrolled proliferative levels in the kidney, interstitial fibrosis is profoundly relevant to renal function as well^27^. Previous studies have demonstrated the relevance between inactivation of AMPK phosphorylation and tissue fibrosis^34^; hence, activating AMPK phosphorylation has been considered a feasible way in anti-fibrosis. No matter the deposition of type IV collagen in the kidneys^48^, or UUO-induced renal fibrosis^49^, even bleomycin-induced pulmonary fibrosis^34^, all could be reversed by activating AMPK phosphorylation as well. Besides, metformin could suppress fibrosis not only through inhibiting the generation of myofibroblasts^50,51^, but also via downregulating the expression of HIF-1α^52^ and TGF-β^27,51^ in either kidney or lung.

In our study, decreased renal fibrosis was detected in metformin-treated *Tsc1*^ptKO^ group, evidenced by reduced α-SMA-positive fibroblasts as well as decreased fibronectin and reduced collagen levels. Our in vitro experiments further demonstrated that the suppression of fibronectin production was dependent on metformin-induced AMPK phosphorylation. The reduced renal interstitial fibrosis by metformin may contribute, at least in part, to the ameliorated renal function. Interestingly, Vesey et al.^53^ once showed increased secretion of α-SMA and fibronectin under IL-1 stimulation in proximal tubule cells, which suggests that the suppressed fibrosis might be relevant to inhibition of inflammation as well.

Besides fibrosis, an elevated level of renal interstitial inflammation is also displayed in *Tsc1*^ptKO^ mice, which could further damage renal function^27^. AMPK phosphorylation, on the contrary, inhibited inflammation by suppressing the expression of IL-6^54^, TNF-α, and MCP-1 in adipocytes^24^, intestinal epithelial cells^22^, or kidney of *db*/*db* mouse^48^. Therefore, the reactivation of AMPK phosphorylation is likely to be a promising anti-inflammation strategy for the treatment of TSC-associated renal diseases. As a potent AMPK activator, metformin was considered powerful in reducing inflammation, which not only protected renal function in DN^55^ or attenuated renal injuries induced by cyclophosphamide^44^, but even slowed the progression of atherosclerosis as well by activating AMPK phosphorylation^32,33^.

Our results demonstrated significant downregulation of inflammatory cell markers (F4/80, CD3, CD4, TNF-α, IL-1β, and IL-6) as the potential mechanism by which metformin inhibits inflammatory responses through increased AMPK phosphorylation in *Tsc1*^ptKO^ mice. Furthermore, siRNA downregulation of *Tsc1* induced a significant increase of IL-1β production in renal proximal tubular epithelial cells in vitro. Treatment with metformin abolished the increase, which was reversed by the AMKP inhibitor, dorsomorphin. Thus, the potent anti-inflammatory effect of metformin in treating TSC-related renal pathology is dependent on AMPK phosphorylation.

In the present, we report for the first time that metformin can effectively treat the aberrant kidney growth and renal pathology caused by *Tsc1* deletion in the renal proximal tubule cells of *Tsc1*^ptKO^ mice. Mechanistically, our results indicate that metformin treatment inhibits hyperphosphorylation of Akt at both Thr-308 and Ser-473, and thereby prevents activation and aberrant proliferation of the renal proximal tubule cells, thus preventing proteinuria and maintaining kidney function. Importantly, we also observed that metformin treatment additionally inhibits the production of pro-inflammatory cytokines, suppresses infiltration of inflammatory cells, and reduces deposition of fibrotic extracellular matrix proteins in the kidneys of *Tsc1*^ptKO^ mice. These multiple beneficial effects are likely what have conferred the remarkable therapeutic efficacy of metformin for the treatment of *Tsc1* deletion-induced kidney pathology. Our additional mechanistic cell culture studies unveiled that these therapeutic effects of metformin are accompanied by reactivation of AMPK phosphorylation at Thr-172 that is antagonized by the AMPK inhibitor, dorsomorphin. Thus, it is our hope that this report will lead to clinical trials for the treatment of TSC-associated manifestations by repurposing the use of metformin, which is currently prescribed for the treatment of type 2 diabetic patients.

## Supplementary information

Supplementary Figure 1

Supplementary Figure Legends

## References

[1] Henske EP, Jozwiak S, Kingswood JC, Sampson JR, Thiele EA (2016). Tuberous sclerosis complex. Nat. Rev. Dis. Prim..

[2] van Slegtenhorst M (1997). Identification of the tuberous sclerosis gene TSC1 on chromosome 9q34. Science.

[3] European Chromosome 16 Tuberous Sclerosis, C. (1993). Identification and characterization of the tuberous sclerosis gene on chromosome 16. Cell.

[4] Dibble CC (2012). TBC1D7 is a third subunit of the TSC1-TSC2 complex upstream of mTORC1. Mol. Cell.

[5] Wataya-Kaneda M (2017). Tuberous sclerosis complex: recent advances in manifestations and therapy. Int J. Urol..

[6] Rosset C, Netto CBO, Ashton-Prolla P (2017). TSC1 and TSC2 gene mutations and their implications for treatment in tuberous sclerosis complex: a review. Genet. Mol. Biol..

[7] Buj Pradilla MJ, Marti Balleste T, Torra R, Villacampa Auba F (2017). Recommendations for imaging-based diagnosis and management of renal angiomyolipoma associated with tuberous sclerosis complex. Clin. Kidney J..

[8] Bissler JJ, Kingswood JC (2004). Renal angiomyolipomata. Kidney Int..

[9] O’Callaghan FJ, Shiell AW, Osborne JP, Martyn CN (1998). Prevalence of tuberous sclerosis estimated by capture-recapture analysis. Lancet.

[10] Samuels JA (2017). Treatment of renal angiomyolipoma and other hamartomas in patients with tuberous sclerosis complex. Clin. J. Am. Soc. Nephrol..

[11] Wu H (2016). Blocking rpS6 phosphorylation exacerbates Tsc1 deletion-induced kidney growth. J. Am. Soc. Nephrol..

[12] Bee J, Fuller S, Miller S, Johnson SR (2018). Lung function response and side effects to rapamycin for lymphangioleiomyomatosis: a prospective national cohort study. Thorax.

[13] Palavra F, Robalo C, Reis F (2017). Recent advances and challenges of mTOR inhibitors use in the treatment of patients with tuberous sclerosis complex. Oxid. Med. Cell Longev..

[14] Bissler JJ (2008). Sirolimus for angiomyolipoma in tuberous sclerosis complex or lymphangioleiomyomatosis. N. Engl. J. Med..

[15] Yao J (2014). Sustained effects of sirolimus on lung function and cystic lung lesions in lymphangioleiomyomatosis. Am. J. Respir. Crit. Care Med..

[16] Brook-Carter PT (1994). Deletion of the TSC2 and PKD1 genes associated with severe infantile polycystic kidney disease—a contiguous gene syndrome. Nat. Genet..

[17] Serra AL (2010). Sirolimus and kidney growth in autosomal dominant polycystic kidney disease. N. Engl. J. Med..

[18] Walz G (2010). Everolimus in patients with autosomal dominant polycystic kidney disease. N. Engl. J. Med..

[19] Hardie DG, Ross FA, Hawley SA (2012). AMPK: a nutrient and energy sensor that maintains energy homeostasis. Nat. Rev. Mol. Cell Biol..

[20] Winder WW, Hardie DG (1999). AMP-activated protein kinase, a metabolic master switch: possible roles in type 2 diabetes. Am. J. Physiol..

[21] Takiar V (2011). Activating AMP-activated protein kinase (AMPK) slows renal cystogenesis. Proc. Natl Acad. Sci. USA.

[22] Chi JH, Seo GS, Lee SH (2018). Oregonin inhibits inflammation and protects against barrier disruption in intestinal epithelial cells. Int. Immunopharmacol..

[23] Fox MM, Phoenix KN, Kopsiaftis SG, Claffey KP (2013). AMP-activated protein kinase alpha 2 isoform suppression in primary breast cancer alters AMPK growth control and apoptotic signaling. Genes Cancer.

[24] Jung, T. W., Chung, Y. H., Kim, H. C., Abd El-Aty, A. M. & Jeong, J. H. Protectin DX attenuates LPS-induced inflammation and insulin resistance in adipocytes via AMPK-mediated suppression of the NFkappaB pathway. *Am. J. Physiol. Endocrinol. Metab.*10.1152/ajpendo.00408.2017 (2018). 10.1152/ajpendo.00408.2017 29584445

[25] Liu, Y. *et al*. Resveratrol inhibits the proliferation and induces the apoptosis in ovarian cancer cells via inhibiting glycolysis and targeting AMPK/mTOR signaling pathway. *J. Cell. Biochem*. 10.1002/jcb.26822 (2018). 10.1002/jcb.26822 29663499

[26] Lam HC, Siroky BJ, Henske EP (2018). Renal disease in tuberous sclerosis complex: pathogenesis and therapy. Nat. Rev. Nephrol..

[27] Cavaglieri RC, Day RT, Feliers D, Abboud HE (2015). Metformin prevents renal interstitial fibrosis in mice with unilateral ureteral obstruction. Mol. Cell Endocrinol..

[28] Rowe I (2013). Defective glucose metabolism in polycystic kidney disease identifies a new therapeutic strategy. Nat. Med..

[29] van Veelen W, Korsse SE, van de Laar L, Peppelenbosch MP (2011). The long and winding road to rational treatment of cancer associated with LKB1/AMPK/TSC/mTORC1 signaling. Oncogene.

[30] Wu, D. *et al*. Glucose-regulated phosphorylation of TET2 by AMPK reveals a pathway linking diabetes to cancer. *Nature*10.1038/s41586-018-0350-5 (2018). 10.1038/s41586-018-0350-5 PMC6430198 30022161

[31] Daugan M, Dufay Wojcicki A, d’Hayer B, Boudy V (2016). Metformin: an anti-diabetic drug to fight cancer. Pharm. Res..

[32] Yang Q (2018). Metformin ameliorates the progression of atherosclerosis via suppressing macrophage infiltration and inflammatory responses in rabbits. Life Sci..

[33] Di Fusco, D. et al. Metformin inhibits inflammatory signals in the gut by controlling AMPK and p38 MAP kinase activation. *Clin. Sci.*10.1042/CS20180167 (2018). 10.1042/CS20180167 29540537

[34] Rangarajan, S. et al. Metformin reverses established lung fibrosis in a bleomycin model. *Nat. Med*. 10.1038/s41591-018-0087-6 (2018). 10.1038/s41591-018-0087-6 PMC6081262 29967351

[35] Iwano M (2002). Evidence that fibroblasts derive from epithelium during tissue fibrosis. J. Clin. Invest..

[36] Chen J (2012). EGFR signaling promotes TGFbeta-dependent renal fibrosis. J. Am. Soc. Nephrol..

[37] Chen JK, Falck JR, Reddy KM, Capdevila J, Harris RC (1998). Epoxyeicosatrienoic acids and their sulfonimide derivatives stimulate tyrosine phosphorylation and induce mitogenesis in renal epithelial cells. J. Biol. Chem..

[38] Chen J, Chen MX, Fogo AB, Harris RC, Chen JK (2013). mVps34 deletion in podocytes causes glomerulosclerosis by disrupting intracellular vesicle trafficking. J. Am. Soc. Nephrol..

[39] Chen J, Chen JK, Conway EM, Harris RC (2013). Survivin mediates renal proximal tubule recovery from AKI. J. Am. Soc. Nephrol..

[40] Chen JK, Chen J, Thomas G, Kozma SC, Harris RC (2009). S6 kinase 1 knockout inhibits uninephrectomy- or diabetes-induced renal hypertrophy. Am. J. Physiol. Ren. Physiol..

[41] Xu J, Chen J, Dong Z, Meyuhas O, Chen JK (2015). Phosphorylation of ribosomal protein S6 mediates compensatory renal hypertrophy. Kidney Int..

[42] Chen JK, Capdevila J, Harris RC (2001). Cytochrome p450 epoxygenase metabolism of arachidonic acid inhibits apoptosis. Mol. Cell Biol..

[43] Lash LH, Putt DA, Matherly LH (2002). Protection of NRK-52E cells, a rat renal proximal tubular cell line, from chemical-induced apoptosis by overexpression of a mitochondrial glutathione transporter. J. Pharm. Exp. Ther..

[44] Liu Q (2016). Paeoniflorin ameliorates renal function in cyclophosphamide-induced mice via AMPK suppressed inflammation and apoptosis. Biomed. Pharmacother..

[45] Li W, Saud SM, Young MR, Chen G, Hua B (2015). Targeting AMPK for cancer prevention and treatment. Oncotarget.

[46] Zhao Y (2017). ROS signaling under metabolic stress: cross-talk between AMPK and AKT pathway. Mol. Cancer.

[47] Kapodistria, K., Tsilibary, E. P., Kotsopoulou, E., Moustardas, P. & Kitsiou, P. Liraglutide, a human glucagon-like peptide-1 analogue, stimulates AKT-dependent survival signalling and inhibits pancreatic beta-cell apoptosis. *J. Cell. Mol. Med*. 10.1111/jcmm.13259 (2018). 10.1111/jcmm.13259 PMC5980190 29524296

[48] Kim MY (2013). Resveratrol prevents renal lipotoxicity and inhibits mesangial cell glucotoxicity in a manner dependent on the AMPK-SIRT1-PGC1alpha axis in db/db mice. Diabetologia.

[49] Kim H (2015). Activation of AMP-activated protein kinase inhibits ER stress and renal fibrosis. Am. J. Physiol. Ren. Physiol..

[50] Tanjore H (2009). Contribution of epithelial-derived fibroblasts to bleomycin-induced lung fibrosis. Am. J. Respir. Crit. Care Med..

[51] Li L (2015). Metformin attenuates gefitinib-induced exacerbation of pulmonary fibrosis by inhibition of TGF-beta signaling pathway. Oncotarget.

[52] Li X (2016). The role of metformin and resveratrol in the prevention of hypoxia-inducible factor 1alpha accumulation and fibrosis in hypoxic adipose tissue. Br. J. Pharm..

[53] Vesey DA (2002). Interleukin-1beta induces human proximal tubule cell injury, alpha-smooth muscle actin expression and fibronectin production. Kidney Int..

[54] Cui, W. *et al*. Nrf2 attenuates inflammatory response in COPD/emphysema: Crosstalk with Wnt3a/beta-catenin and AMPK pathways. *J. Cell. Mol. Med.*10.1111/jcmm.13628 (2018). 10.1111/jcmm.13628 PMC6010849 29659176

[55] Xie R (2017). The protective effect of betulinic acid (BA) diabetic nephropathy on streptozotocin (STZ)-induced diabetic rats. Food Funct..

