# Supplementary material for: Metformin effectively treats Tsc1 deletion-caused kidney pathology by upregulating AMPK phosphorylation
Source: Cell Death Discov. 2020 Jun 15;6:52. doi: 10.1038/s41420-020-0285-0 (PMC7295815; doi:10.1038/s41420-020-0285-0)
Supplement: Supplementary file 2 — Supplementary Figure Legends [file 41420_2020_285_MOESM2_ESM.docx]

**Figure S1. Generation of *Tsc1* gene-deficient mice**

(A) A schematic depicting genetic deletion of renal proximal tubule-specific *Tsc1* knockout (*Tsc1*^ptKO^) mice, resulting in *Tsc1* gene-deficient (*Tsc1*^ptKO^) mice and *Tsc1*^Ctrl^ littermates, which was used as control for *Tsc1* deletion. (B) Genotyping of *Tsc1* knockout (*Tsc1*^ptKO^) mice. Left image: Electrophoresis of *Tsc1* mutant (230 bp), wild type (193 bp) genotypes. Right image: Electrophoresis of *γ*GT-Cre (370 bp) genotypes. Representative (C) mice and (D) kidney images of indicated two genotypes of mice by 4 weeks of age.
